# Reporting Bias in Drug Trials Submitted to the Food and Drug Administration: Review of Publication and Presentation

**DOI:** 10.1371/journal.pmed.0050217

**Published:** 2008-11-25

**Authors:** Kristin Rising, Peter Bacchetti, Lisa Bero

**Affiliations:** 1 School of Medicine, University of California San Francisco, San Francisco, California, United States of America; 2 Department of Epidemiology and Biostatistics, University of California San Francisco, San Francisco, California, United States of America; 3 Clinical Pharmacy and Health Policy, University of California San Francisco, San Francisco, California, United States of America; University of Ioannina, Greece

## Abstract

**Background:**

Previous studies of drug trials submitted to regulatory authorities have documented selective reporting of both entire trials and favorable results. The objective of this study is to determine the publication rate of efficacy trials submitted to the Food and Drug Administration (FDA) in approved New Drug Applications (NDAs) and to compare the trial characteristics as reported by the FDA with those reported in publications.

**Methods and Findings:**

This is an observational study of all efficacy trials found in approved NDAs for New Molecular Entities (NMEs) from 2001 to 2002 inclusive and all published clinical trials corresponding to the trials within the NDAs. For each trial included in the NDA, we assessed its publication status, primary outcome(s) reported and their statistical significance, and conclusions. Seventy-eight percent (128/164) of efficacy trials contained in FDA reviews of NDAs were published. In a multivariate model, trials with favorable primary outcomes (OR = 4.7, 95% confidence interval [CI] 1.33–17.1, *p* = 0.018) and active controls (OR = 3.4, 95% CI 1.02–11.2, *p* = 0.047) were more likely to be published. Forty-one primary outcomes from the NDAs were omitted from the papers. Papers included 155 outcomes that were in the NDAs, 15 additional outcomes that favored the test drug, and two other neutral or unknown additional outcomes. Excluding outcomes with unknown significance, there were 43 outcomes in the NDAs that did not favor the NDA drug. Of these, 20 (47%) were not included in the papers. The statistical significance of five of the remaining 23 outcomes (22%) changed between the NDA and the paper, with four changing to favor the test drug in the paper (*p* = 0.38). Excluding unknowns, 99 conclusions were provided in both NDAs and papers, nine conclusions (9%) changed from the FDA review of the NDA to the paper, and all nine did so to favor the test drug (100%, 95% CI 72%–100%, *p* = 0.0039).

**Conclusions:**

Many trials were still not published 5 y after FDA approval. Discrepancies between the trial information reviewed by the FDA and information found in published trials tended to lead to more favorable presentations of the NDA drugs in the publications. Thus, the information that is readily available in the scientific literature to health care professionals is incomplete and potentially biased.

## Abstract 2

Lisa Bero and colleagues review the publication status of all efficacy trials carried out in support of new drug approvals from 2001 and 2002, and find that a quarter of trials remain unpublished.

## Editors' Summary

**Background.:**

All health-care professionals want their patients to have the best available clinical care—but how can they identify the optimum drug or intervention? In the past, clinicians used their own experience or advice from colleagues to make treatment decisions. Nowadays, they rely on evidence-based medicine—the systematic review and appraisal of clinical research findings. So, for example, before a new drug is approved for the treatment of a specific disease in the United States and becomes available for doctors to prescribe, the drug's sponsors (usually a pharmaceutical company) must submit a “New Drug Application” (NDA) to the US Food and Drug Administration (FDA). The NDA tells the story of the drug's development from laboratory and animal studies through to clinical trials, including “efficacy” trials in which the efficacy and safety of the new drug and of a standard drug for the disease are compared by giving groups of patients the different drugs and measuring several key (primary) “outcomes.” FDA reviewers use this evidence to decide whether to approve a drug.

**Why Was This Study Done?:**

Although the information in NDAs is publicly available, clinicians and patients usually learn about new drugs from articles published in medical journals after drug approval. Unfortunately, drug sponsors sometimes publish the results only of the trials in which their drug performed well and in which statistical analyses indicate that the drug's improved performance was a real effect rather than a lucky coincidence. Trials in which a drug did not show a “statistically significant benefit” or where the drug was found to have unwanted side effects often remain unpublished. This “publication bias” means that the scientific literature can contain an inaccurate picture of a drug's efficacy and safety relative to other therapies. This may lead to clinicians preferentially prescribing newer, more expensive drugs that are not necessarily better than older drugs. In this study, the researchers test the hypothesis that not all the trial results in NDAs are published in medical journals. They also investigate whether there are any discrepancies between the trial data included in NDAs and in published articles.

**What Did the Researchers Do and Find?:**

The researchers identified all the efficacy trials included in NDAs for totally new drugs that were approved by the FDA in 2001 and 2002 and searched the scientific literature for publications between July 2006 and June 2007 relating to these trials. Only three-quarters of the efficacy trials in the NDAs were published; trials with favorable outcomes were nearly five times as likely to be published as those without favorable outcomes. Although 155 primary outcomes were in both the papers and the NDAs, 41 outcomes were only in the NDAs. Conversely, 17 outcomes were only in the papers; 15 of these favored the test drug. Of the 43 primary outcomes reported in the NDAs that showed no statistically significant benefit for the test drug, only half were included in the papers; for five of the reported primary outcomes, the statistical significance differed between the NDA and the paper and generally favored the test drug in the papers. Finally, nine out of 99 conclusions differed between the NDAs and the papers; each time, the published conclusion favored the test drug.

**What Do These Findings Mean?:**

These findings indicate that the results of many trials of new drugs are not published 5 years after FDA approval of the drug. Furthermore, unexplained discrepancies between the data and conclusions in NDAs and in medical journals are common and tend to paint a more favorable picture of the new drug in the scientific literature than in the NDAs. Overall, these findings suggest that the information on the efficacy of new drugs that is readily available to clinicians and patients through the published scientific literature is incomplete and potentially biased. The recent introduction in the US and elsewhere of mandatory registration of all clinical trials before they start and of mandatory publication in trial registers of the full results of all the predefined primary outcomes should reduce publication bias over the next few years and should allow clinicians and patients to make fully informed treatment decisions.

**Additional Information.:**

Please access these Web sites via the online version of this summary at http://dx.doi.org/10.1371/journal.pmed.0050217.

- This study is further discussed in a *PLoS Medicine* Perspective by An-Wen Chan
- *PLoS Medicine* recently published a related article by Ida Sim and colleagues: Lee K, Bacchetti P, Sim I (2008) Publication of clinical trials supporting successful new drug applications: A literature analysis. PLoS Med 5: e191. doi:10.1371/journal.pmed.0050191
- The Food and Drug Administration provides information about drug approval in the US for consumers and for health-care professionals; detailed information about the process by which drugs are approved is on the Web site of the FDA Center for Drug Evaluation and Research (in English and Spanish)
- NDAs for approved drugs can also be found on this Web site
- The ClinicalTrials.gov Web site provides information about the US National Institutes of Health clinical trial registry, background information about clinical trials, and a fact sheet detailing the requirements of the FDA Amendments Act 2007 for trial registration
- The World Health Organization's International Clinical Trials Registry Platform is working toward setting international norms and standards for the reporting of clinical trials (in several languages)

## Introduction

Evidence-based clinical medicine relies on the publication of high-quality data to determine standards of patient care. Publication bias occurs when some types of results (e.g., those that are statistically significant) are reported more frequently or more quickly than others [1–4]. Publication bias favors the dissemination of information about clinical interventions showing statistically significant benefit. Publication bias, therefore, may lead to preferential prescribing of newer and more expensive treatment choices and may underestimate the harms of drugs that have been in use for only a limited time, and clinical decisions may be based on erroneous information [5]. The objective of this study is to assess the extent and nature of publication bias among newly approved drugs by determining the publication rate of efficacy trials submitted to the United States Food and Drug Administration (FDA) in approved New Drug Applications (NDAs) for NMEs (New Molecular Entities) and comparing the trial characteristics as reported by the FDA with those reported in publications.

Publication bias not only limits the number and scope of studies available for review by clinicians, but also affects the results of systematic reviews and meta-analyses. Researchers may estimate spuriously large treatment effects in early meta-analyses of the available evidence if there is publication bias [4].

The fact that unpublished trials are hidden from view makes it difficult to study publication bias among drug trials [6–8]. However, drug manufacturers seeking approval to market drugs in the US are required to submit all studies to the FDA as part of their NDA. Thus, documents submitted to regulatory agencies can be used to help identify unpublished trials. Past studies found selective reporting of both entire trials and favorable results [9–13]. One study conducted in the US examined 37 trials of nonsteroidal anti-inflammatory drugs that were submitted to the FDA and found that only one trial was published [14]. However, because only one trial was published, trial characteristics that might be associated with publication could not be examined. Another US study examined trials of antidepressants that were submitted to the FDA and also found evidence of publication bias, although this analysis was again limited to one drug category and included drugs approved in a wide range of years (1987–2004), during which time publication practices may have changed substantially as journals began to require trial registration [12].

In this study, we hypothesized that not all data submitted to the FDA for a new drug approval are published and that certain trial characteristics may be associated with publication. We also hypothesized that there are discrepancies between trial data submitted to the FDA and data found in published trials. We compared the efficacy trials submitted to the FDA in approved NDAs with their publications, if any, in the medical literature. For those trials that were published, we compared characteristics of each trial as reported by the FDA with characteristics as reported in the corresponding publication.

## Methods

We conducted a study of all efficacy trials found in approved NDAs for new molecular entities (NMEs) from 2001 to 2002 inclusive and all published clinical trials corresponding to the trials within the NDAs. NMEs are a subset of NDAs that represent novel active ingredients rather than, for example, “me-too” drugs, which are very similar to existing drugs, or combinations of previously approved drugs. Thus, NMEs are clinically important drugs about which new information is needed.

### Search Strategy: New Drug Applications (NDAs)

We (KR and LB) first identified all NDAs for NMEs approved from January 2001 to December 2002. An NME, as defined by the FDA, is an active ingredient that has never before been marketed in the US in any form [15]. We found this information at the Center for Drug Evaluation and Research website [16].

This 2-year period was selected in order to include trials that were recently conducted, while still allowing enough time for publication. Prior research found a median time to publication of 5.5 y from the time a trial started enrollment, with trials with positive results published significantly earlier than trials with negative results (4.3 y versus 6.5 y, respectively) [4]. NDAs for drugs that were withdrawn from the market were excluded, as withdrawal of the drug would likely affect the subsequent publication of trials.

Information submitted in NDAs is contained in the FDA Medical and Statistical Officer Reviews of each NDA, which are publicly available, along with approval letters, at http://www.accessdata.fda.gov/scripts/cder/drugsatfda/index.cfm?fuseaction=Search.Search_Drug_Name.

### Search Strategy: NDA Trials

The approval letter for each drug was read to determine the indication(s) for which the drug was approved. The medical and statistical officer reviews were then reviewed to identify all clinical trials referred to within efficacy sections of the reviews that met our inclusion criteria. We call these trials that were submitted in support of efficacy “efficacy trials.”

### Inclusion Criteria for Trials within the NDA

Each NDA submitted to the FDA contains one or more efficacy trials for each indication for which the drug is under evaluation. We included in our study all trials that the FDA officers included in their efficacy review of the NDA for the approved indication(s) only.

Specific inclusion criteria for trials within the FDA reviews were: had a comparison group (placebo or comparator drug); included patients with the condition for which the NDA was approved; were used by the FDA in the efficacy review of the drug; had separately reported results somewhere within the medical or statistical review, unless a pooled analysis of two or more trials was pre-specified; were reported with enough information to be able to search for them within the published literature (for example, some combination of patient population description, intervention description, sample size, outcomes measured, location of trial, etc.); and had complete analysis at time of reporting, or analysis of the primary outcome(s) was complete if the trials had long-term extensions.

### Search Strategy: Published Trials

We performed an electronic search of PubMed and The Cochrane Library from July 2006 through June 2007, searching on MESH terms and keywords of drug names, to identify publications that corresponded to each of the trials identified in the FDA reviews of each NDA. Trade and generic names were used to search online databases, as well as any other names, such as code names, that had been used in the NDA text describing the trial. We searched for articles published in any language. In addition, the reference lists of identified articles were scanned for additional relevant published trials and investigators and drug companies were contacted multiple times to inquire about publication status.

If no publications were identified for a trial within a specific NDA, authors of other publications identified for that drug were contacted to request assistance identifying the publications of the other trials. Trials were referred to as protocol numbers when available from the NDA, and otherwise by name or any other specific identifying information (such as location of study, number of participants, or intervention groups). If further assistance was required, the drug company was contacted. If a publication was identified that was thought to possibly be one of the included trials, but the identity was not certain, the author of that publication was contacted to inquire whether the publication was reporting the trial of interest (identified by protocol number if available). When contacting authors, the corresponding author was contacted first. If the author did not reply, a second email was sent or a phone call was made if a phone number was available. Attempts were also made to contact any other authors of the publications for whom contact information could be found. On average, about three attempts were made to establish contact with someone (author or drug company) regarding publication of each trial. This process was followed with each publication identified for each trial included in the study. Contact was made only in instances in which trial identity was unclear in a publication, or for which no publication had been found for a trial.

Published trials were linked to trials found in the NDA by comparing descriptions of methods and data on the characteristics of the population studied, including sample size, intervention groups, outcomes measured, and results of the trial.

The publication status of each NDA trial was recorded as (1) individual publication, (2) publication in press, (3) pooled publication reported separately, (4) pooled publication not reported separately, (5) abstract, (6) poster, (7) not published, verified; or (8) no information found. A trial was considered “not published, verified” (category 7 above) only with verification from the drug company or an investigator associated with that trial. Trials that were reported to be “in press” were considered published but were excluded from all additional analysis, as the published reports could not be reviewed.

For analysis, the coding categories above were dichotomized as “published” or “not published”. A trial was coded as “published” if it was in categories 1, 2 or 3 above; it was coded as an individual publication if one or more publications were identified reporting only the results of that specific trial; as in press; or as a pooled publication if multiple trials were included but the results of the NDA trial were reported separately. All other trials (categories 4, 5, 6, 7 or 8 above) were coded as “not published”.

### Data Extraction

We designed a database into which we directly entered all information on publication status and trial characteristics. The database was pre-tested on trials and papers that were not included in our study, and unclear/unreliable data extraction elements were eliminated or revised.

One author (KR) extracted all data from all NDA trials and publications. A random number generator was used to determine the order in which the drugs were reviewed. The data from all trials, both in the NDA and publications, were coded before moving on to the next drug. Additional coders were trained to double-code portions of the data determined to be potentially subjective in both the FDA reviews and the publications (see Box 1). Double-coding was done in a separate random order and was performed for all papers (*n* = 126) and for a randomly selected subset of NDA trials (*n* = 55). Disagreements were resolved by consensus. Because previous research suggests that masking of articles to reviewers does not influence ratings, reviewers were not be blinded to the source or authors of the articles [17–19]. Inter-coder reliability was very good (simple kappa 0.71 to 1).

We collected data on characteristics of randomized controlled trials that could influence bias including randomization [20,21], double blinding [21,22], choice of drug comparator [23–26], peer-review status of the publication [27], funding source [28,29], and financial ties of investigators [29,30] because these are characteristics that could be associated with publication (see Box 1 for data coding). Details on the collection of outcomes and conclusions, as well as publication status, are described below.

#### Primary outcome(s) (double-coded).

The primary outcome(s), as identified in the FDA review of the NDA or the publication, were recorded for each trial. If a primary outcome was measured at different times, we used the time point that was specified in the NDA as the primary outcome.

If a primary outcome was not explicitly stated, the outcome used in the power analysis was designated as the primary outcome. If the power analysis was not provided or an outcome was not stated, then the outcome stated in the primary research question was designated as primary. If a primary outcome was not identified by any of these methods, the trial was considered to have no primary outcomes reported.

For each primary outcome, we recorded: definition of the outcome, whether the outcome measured efficacy or safety, and the statistical significance of the outcome. Statistical significance of the outcome was coded as 1) statistically significant in favor of the NDA drug (e.g., *p* < 0.05, 95% CI for difference excluding 0, or 95% CI for ratio excluding 1), 2) not statistically significant, 3) statistically significant in favor of the comparator or 4) unknown. For trials with only one primary outcome, the outcome was considered favorable if it was a superiority outcome that was statistically significant in favor of the NDA drug or in which equivalence was found. Superiority outcomes that were not statistically significant or that favored the comparator were considered not favorable, and outcomes for which the significance was not reported were excluded (13 out of 196 outcomes).

For trials with multiple primary outcomes, a composite field was made and was coded on the basis of the statistical significance of each primary outcome in the trial. The composite outcome was coded as follows: (1) favorable: at least one primary outcome was statistically significant in favor of the drug and no primary outcomes were statistically significant in favor of the comparator or (2) not favorable: no primary outcomes were statistically significant in favor of the NDA drug or at least one primary outcome was statistically significant in favor of the comparator.

#### Conclusion (double-coded).

The conclusion for each trial, as stated in the FDA review of the NDA or publication, was recorded as (1) favorable to the test drug, (2) neutral, (3) not favorable to the test drug, or (4) unknown/not stated.

#### Publication status.

The publication status of each NDA trial was recorded as (1) individual publication, (2) publication in press, (3) pooled publication reported separately, (4) pooled publication not reported separately, (5) abstract, (6) poster, (7) not published, verified, or (8) no information found.

For analysis, the coding categories above were dichotomized as “published” or “not published.” A trial was coded as “published” if it was in categories 1, 2, or 3 above; it was coded as an individual publication if one or more publications were identified reporting only the results of that specific trial; as in press; or as a pooled publication if multiple trials were included but the results of the NDA trial were reported separately. All other trials (categories 4, 5, 6, 7 or 8) were considered “not published.”

In cases of duplicate publications, the reports were reviewed to identify the more complete publication, and only this publication was included in the analysis. In many cases, publications were identified as post-hoc analyses or reports of secondary variables. If the identity of the primary publication was unclear, the authors were contacted and asked to identify the primary publication.

### Statistical Analysis

For our primary outcome of publication, we report the publication status of NDA trials in terms of the whole population of trials (the percentage of trials published) as well as per NDA (the percentage of NDAs with at least one published trial). We report the frequency of the different characteristics of each trial as reported in both the FDA reviews of the NDA and the publication (see Box 1 for list of characteristics). For characteristics for which there was sufficient variability, we assessed whether there was an association with publication.

Associations between individual trial characteristics as reported in the NDA and publication were first analyzed using univariate logistic regression, with a random effect included to account for possible similarity of trials from the same NDA, and odds ratios were estimated. Multivariate models were then built in a forward stepwise manner until no remaining candidate predictors had *p* < 0.05.

For the subset of published trials, we compared the characteristics of the NDA trials with their corresponding publication. We tested for systematic changes in the reporting of each trial characteristic from the individual NDA report to the published report using McNemar's test for dichotomous variables and the paired *t*-test for numeric variables.

We identified primary outcomes that were added or deleted from papers and report the statistical significance of these outcomes. We also report changes in statistical significance of primary outcomes from the NDA to publication.

We report changes in conclusions from the NDA to publication.

**Box 1.**

Coding of Study Design and Publication Characteristics

*Study design (single-coded; coded for NDA trials only)*

Randomization: Recorded as (1) yes or (2) no/unknown.

Blinding: Recorded as (1) single blind, (2) double blind, (3) triple blind, or (4) open label (not blinded).

*Study design (double-coded; coded for NDA trials and publications)*

Intervention groups: Details for each intervention group were recorded, including: drug name, dose, frequency, route, duration, and whether the drug was an NDA drug, comparator drug or placebo.

Intention to treat (ITT): Recorded as (1) yes or (2) no/unknown. Each trial was coded as using ITT analysis if the number of patients for the primary efficacy analysis equaled the number of patients randomized. If use of ITT was stated in the methods, but randomized patients were omitted from the primary efficacy population, then the field was coded as “ITT not done, but report states that it was done.” We applied the following definition of ITT: “All participants should be included regardless of whether their outcomes were actually collected.” [37].

Last observation carried forward (LOCF): Recorded as (1) yes or (2) no/unknown

Data imputation: Recorded as (1) yes or (2) no/unknown. This included those trials that used LOCF.

Data reporting: Reporting of raw data (e.g., counts or means with no statistical manipulation, such as, number of patients with outcome, actual blood pressure as opposed to change in blood pressure), percent data (e.g., X% of patients responded), absolute change, relative risk, and number need to treat (NNT) for the primary outcome(s) of each trial was recorded.

Uncertainty reporting: Reporting of *p*-values, confidence intervals, and standard deviation for the primary outcome(s) of each trial was recorded.

Adverse events table: Each trial was assessed for the presence of an adverse event table. Details of statistically significant adverse events were also recorded.

*Publication characteristics (single-coded; coded for publications only)*

Funding: The funding source(s), as disclosed in each paper, were recorded as (1) industry, (2) government, (3) non-profit, or (4) not disclosed. The specific funding source(s) were also recorded.

Disclosure of conflicts: Disclosure of conflicts in each paper was recorded as (1) individual conflicts disclosed (details recorded under each author), (2) conflicts not disclosed, (3) employees only disclosed, or (4) “no conflicts” explicitly stated.

Author affiliation with industry: Recorded as (1) yes or (2) no/unknown. Details of any affiliation with industry (such as employment by industry) were recorded for each author, as disclosed in the publication.

Peer-review status of journal: Each journal article was classified as peer-reviewed, not peer-reviewed or unknown status. The editorial office of each journal was contacted to determine whether the journal was peer reviewed and, if peer-reviewed, how long a peer review process had been in place to ensure that earlier publications were included in the peer review process. Websites were also consulted for those journals for which the editorial office did not reply.

Impact factor: The impact factor was obtained for each journal for the year in which each article was published from the Institute for Scientific Information website [38]. Impact factors that were not found at the ISI website (articles published before 2000) were found in the UCSF library microfilms in the Science Citation Index Journal Citation Reports from the Institute for Scientific Information.

## Results

### Characteristics of Included Trials from NDAs

**Figure 1:**
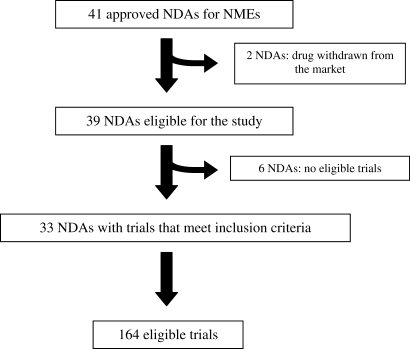
Flow Chart for Selection of New Drug Applications and Included Trials

Our final sample consisted of 164 efficacy trials found in 33 NDAs (see Figure 1). The NDAs were supported by a range of 1–13 trials with a median of four trials. See Table S1 for list of NDAs and included trials.

Table 1 shows the characteristics of the full sample of trials, by publication status. All trials were reported as randomized except for one, for which randomization was not mentioned. There were approximately equal proportions of active- and placebo-controlled trials. Only 14% (23/164) of trials used intention to treat (ITT) analysis as their primary analysis. Furthermore, the definition of ITT that we used (see Box 1) includes per-protocol analysis (in which participants are analyzed as treated rather than as randomized). Thus, an even lower proportion of trials adhered to ITT analysis defined as all participants included in the arm to which they were allocated, whether or not they received (or completed) the intervention given to that arm. Another 53% (74/141) stated that they conducted ITT analysis, although their primary analysis did not include all randomized patients. Uncertainty was most frequently reported as a *p* value (81%, 132/163), although 45% (73/163) of trials reported confidence intervals. The primary outcome composite field was favorable to the test drug in 76% (124/163) of the trials. The conclusion favored the test drug in 70% (114/164), was neutral in 7% (11/164), was not favorable to the test drug in 4% (7/164), and was unknown in 20% (32/164) of trials.

**Table 1:**
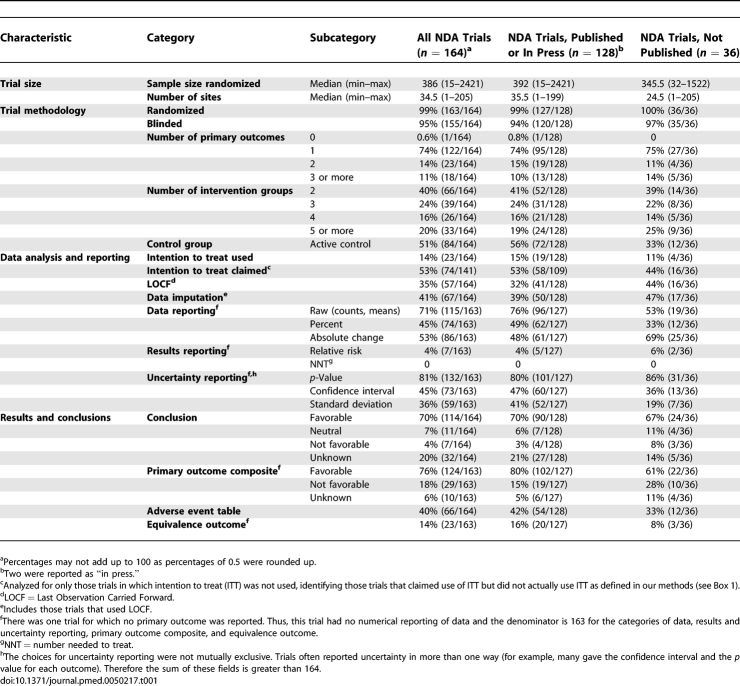
Characteristics of Included NDA Trials by Publication Status (*n* = 164)

### Publication Status

As shown in Table 1, 77% (128/164) trials were published, which included 100 individual publications, 26 pooled publications, and two in-press publications. The two trials that were in press were omitted from all subsequent analyses comparing NDA trials with publications. Of the 36 trials that were not published, 15 were incompletely published in either a pooled publication or an abstract and 12 were verified as not published. Information for nine trials could not be found, even after multiple contacts with the drug companies and authors of other publications.

The NDAs had a range of one to 13 published trials, with 94% (31/33) of NDAs having at least one trial published, and 52% (17/33) of NDAs having all trials published. There were two NDAs (dutasteride and perflutren), with a total of five trials, for which no trials were published (see Table S1).

We did not identify any specific cases in which sponsors prohibited investigators from publishing. However, some investigators did comment that they had been eager to publish their findings. As one investigator stated, “The data are in my opinion very worthwhile. Efforts were made a number of times to work on publishing the data, but it was never possible to find a time when both the PI and the company simultaneously had time available to commit.” Another commented, “Unfortunately I do not think this complete study has ever been published. It is clearly important that this should be published. I have been and continue to be in contact with [Company] to see how this can be published.”

### Predictors of Publication

Table 2 shows the results of univariate logistic regression analyses, which found that trials that were reported in the NDA as having active controls or with favorable primary outcomes were significantly more likely to be published. In a multivariate model, both active control and favorable primary outcome remained significant. No other variable had *p* < 0.05 when added to the model with those two predictors. Although favorable conclusion had a sizeable estimated odds ratio in the univariate model, this disappeared when controlling for active controls and favorable primary outcomes (OR = 0.99, 95% CI 0.15 to 6.4, *p* = 0.99).

**Table 2:**
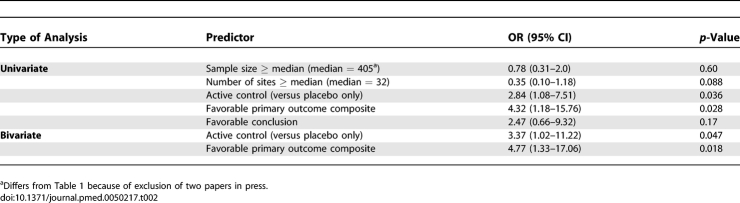
Predictors of Publication: Univariate and Bivariate Analysis

### Discrepancies between Data Analysis and Reporting in the NDA and Published Trials

As shown in Table 3, there were statistically significant changes in the reporting of types of data, *p* values, confidence intervals, and adverse event tables in the publications as compared with the NDAs.

**Table 3:**
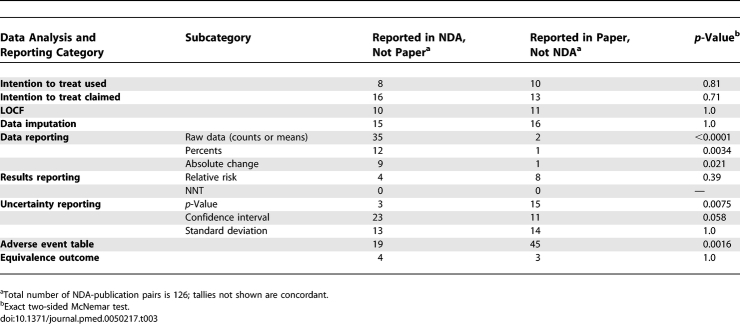
Numbers of Trials with Discrepancies Concerning Data Analysis and Reporting Between the NDAs and the Published Trials

### Discrepancies in Primary Outcome Reporting

As shown in Table 4, trials had a total of 179 primary outcomes reported in the NDAs. Forty-one primary outcomes from the NDAs were omitted from the papers. Papers included 155 outcomes that were also in the NDAs (87%, 155/179), plus 15 additional outcomes that favored the test drug, and two other neutral or unknown additional outcomes. Thus, the papers included more outcomes favoring the test drug than did the NDAs.

**Table 4:**
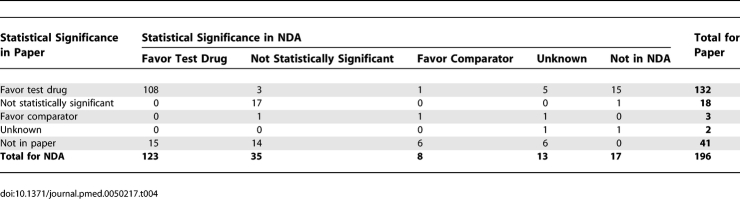
Numbers of Primary Outcomes with Concordance or Changes in Reporting from NDA to Publication

Excluding outcomes with unknown significance, there were 43 outcomes in the NDAs that did not favor the test drug (35 not statistically significant, eight favored the comparator). Of these outcomes, 20 (47%) were not included in the papers. In addition, the statistical significance of five of the remaining 23 outcomes (22%) changed between the NDA and the paper, with four changing to favor the test drug in the paper (*p* = 0.38). The changes in outcomes occurred in a total of 36 trials found in 19 different NDAs.

### Discrepancies in Conclusions

As shown in Table 5, when excluding unknown conclusions, 99 conclusions were provided in both NDAs and papers. There were ten conclusions in the NDAs that did not favor the test drug (six neutral and four favoring the comparator). Excluding unknowns, nine conclusions (9%) changed from the FDA review of the NDA to the paper, and all nine did so to favor the test drug (100%, 95% CI 72% to 100%, *p* = 0.0039). Including the unknowns, 35 of 36 that changed did so to favor the test drug (*p* < 0.0001).

**Table 5:**
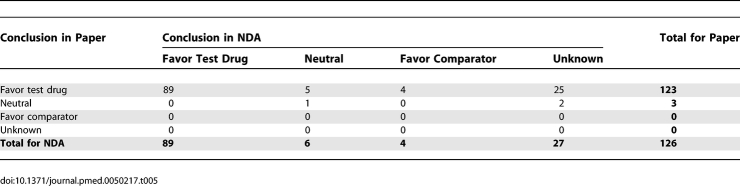
Numbers of Trials with Concordance or Changes in Reporting of Conclusion from NDA to Publication

The changes in statistical significance of outcomes and conclusions were too few to permit a meaningful investigation of predictors.

### Characteristics of Publications

Peer review was confirmed for 93% (117/126) of publications, was confirmed as not done for 3% (4/126) of publications, and was unknown for the other five. Most publications (*n* = 102) were in journals that had an impact factor, with a median of 4.14 (range 0.85–34.83). Reporting of conflicts of the individual authors varied, with 57% (72/126) reporting only whether authors were employees of companies, and 7% (9/126) not reporting conflicts at all. Only 4% (5/126) specifically stated that the authors did not have any conflicts. At least one author was affiliated with industry in 80% (101/126), and all authors were reported as affiliated with industry in 15% (19/126) of publications. The majority of publications (77%, 97/126) reported funding, at least in part, by industry.

## Discussion

Publication bias can occur in several ways, including not publishing data at all, selectively reporting data, or framing data. We found evidence of both lack of publication and selective reporting of data. Seventy-eight percent of the trials submitted to the FDA were published, and trials with active controls or statistically significant outcomes favoring the test drug were more likely to be published. In a multivariate model, trials with favorable primary outcomes (OR = 4.7, 95% CI 1.33 to 17.1, p = 0.018) and active controls (OR = 3.4, 95% CI 1.02 to 11.2, p = 0.047) were more likely to be published. In addition, reporting sometimes differed between trials as they were described in FDA reviews and their corresponding publications. These changes included the addition or deletion of outcomes, changes in statistical significance of reported outcomes, and changes in overall trial conclusions. Papers included 155 outcomes that were also in the NDAs, 15 additional outcomes that favored the test drug, and two other neutral or unknown additional outcomes. Excluding outcomes with unknown significance, there were 43 outcomes in the NDAs that did not favor the NDA drug. Of these, 20 (47%) were not included in the papers. The statistical significance of five of the remaining 23 outcomes (22%) changed between the NDA and the paper, with four changing to favor the test drug in the paper (*p* = 0.38). Excluding unknowns, 99 conclusions were provided in both NDAs and papers, nine conclusions (9%) changed from the FDA review of the NDA to the paper, and all nine did so to favor the test drug (100%, 95% CI 72% to 100%, *p* = 0.0039). All of these changes in reporting led to more favorable presentations of the NDA drug in the published articles.

Our findings extend those of others by demonstrating that reporting bias occurs across a variety of drug categories and that statistical significance of reported primary outcomes sometimes changes to give a more favorable presentation in the publications [10–12]. These changes occur primarily in peer-reviewed, moderate impact factor journals that disclose funding sources and other financial ties. Thus, publication of trial data in peer-reviewed publications appears to be inadequate, supporting the need for reporting of full protocols and findings in a trial registry [31–34]. There have been several advances in trial registration, including The World Health Organization International Clinical Trials Registry Platform, a US register (http://www.clinicaltrials.gov), and the International Committee of Journal Editors requirement for pre-registration of all trials published in participating journals from late 2005, and the FDA Amendments Act requirements for study results posting. Our findings suggest that these registries should contain, at a minimum, full reporting of results for all primary outcomes that are reported for trials submitted for regulatory approval.

Responses from investigators to our inquiries about unpublished studies suggest that studies were not published because they were not submitted to journals. Several other studies, based on self-reports from authors with unpublished studies, suggest that authors' decisions not to submit manuscripts account for the majority of unpublished studies [2,7,8,35]. A prospective cohort study of 1107 manuscripts submitted to three major medical journals found that having statistically significant results did not improve the chance of a study being published, although studies of higher methodological rigor were more likely to be published [36]. In our opinion, investigators have an ethical obligation to submit the results of their research for publication.

Our study has several limitations. We were unable to determine why results were changed from the FDA review of the trial in the NDA to publication. Possible explanations for changes in the primary outcome(s) include: there was a problem with the measurement of the primary outcome as identified by the sponsor or the FDA; the primary outcome as submitted to the FDA did not have a favorable result; or the primary outcome was criticized by the FDA. Changes in the reporting of the significance of primary outcome(s) may have been due to either changes in analysis initiated by the sponsor in order to improve the results or changes in analysis due to criticism by the FDA of the initial analysis used. We could not investigate predictors of change to more favorable results because almost all of the results started out as favorable, therefore leaving only a small subset of results that could change to more favorable reporting in the publication. Another limitation was the quality of our data sources from the FDA. Although clinical trial data submitted to the FDA are publicly available, they are not in a format that is easily accessible, and the documents are often incomplete. Trials that are included in the efficacy or safety analysis are not clearly indicated. They could, for example, be listed in a table. In addition, the data are available only in the FDA review, and not as originally submitted by the sponsor. Thus, the primary outcomes as identified in the FDA review could differ from those listed in the original trial protocol. Lastly, it is possible that sponsors may not submit all their data to the FDA.

The goal of this study was to determine whether information that is available to the FDA is readily accessible to clinicians, and whether it is presented in the same way. As we hypothesized, not all data submitted to the FDA in support of a new drug approval were published, and there were discrepancies between original trial data submitted to the FDA and data found in published trials. Thus, the information that is readily available in the scientific literature to health care professionals is incomplete and potentially biased.

## Supporting Information

Table S1 List of NDAs (78 KB DOC)

## Abbreviations

CI: confidence interval
FDA: Food and Drug Administration
ITT: intention to treat
LOCF: last observation carried forward
NDA: New Drug Application
NME: New Molecular Entity
NNT: number need to treat
OR: odds ratio
