# Correction: Reporting Bias in Drug Trials Submitted to the Food and Drug Administration: Review of Publication and Presentation

**DOI:** 10.1371/journal.pmed.1000017

**Published:** 2009-01-27

**Authors:** Kristin Rising, Peter Bacchetti, Lisa Bero

Correction for:

Rising K, Bacchetti P, Bero L (2008) Reporting bias in drug trials submitted to the Food and Drug Administration: Review of publication and presentation. PLoS Med 5(11): e217. doi:10.1371/journal.pmed.0050217

The Results section of this manuscript contains three numerical errors. Under the heading “Discrepancies in Primary Outcome Reporting,” “155 outcomes” should read “138 outcomes” and “155/179” should read “138/179.” Under the heading “Discrepancies in Conclusions,” “35 of 36” should read “34 of 36.”

Please note that these errors were only in the text of the Results section, and not in the detailed results given in Tables 4 and 5, which were correct. The complete and correct text for these two sections can be found in Corrected Results below.

**Corrected Results**

**Discrepancies in Primary Outcome Reporting**

As shown in Table 4, trials had a total of 179 primary outcomes reported in the NDAs. Forty-one primary outcomes from the NDAs were omitted from the papers. Papers included 138 outcomes that were also in the NDAs (77%, 138/179), plus 15 additional outcomes that favored the test drug, and two other neutral or unknown additional outcomes. Thus, the papers included more outcomes favoring the test drug than did the NDAs. Excluding outcomes with unknown significance, there were 43 outcomes in the NDAs that did not favor the test drug (35 not statistically significant, eight favored the comparator). Of these outcomes, 20 (47%) were not included in the papers. In addition, the statistical significance of five of the remaining 23 outcomes (22%) changed between the NDA and the paper, with four changing to favor the test drug in the paper (*p* = 0.38). The changes in outcomes occurred in a total of 36 trials found in 19 different NDAs.

**Discrepancies in Conclusions**

As shown in Table 5, when excluding unknown conclusions, 99 conclusions were provided in both NDAs and papers. There were ten conclusions in the NDAs that did not favor the test drug (six neutral and four favoring the comparator). Excluding unknowns, nine conclusions (9%) changed from the FDA review of the NDA to the paper, and all nine did so to favor the test drug (100%, 95% CI 72% to 100%, *p* = 0.0039). Including the unknowns, 34 of 36 that changed did so to favor the test drug (*p* < 0.0001). The changes in statistical significance of outcomes and conclusions were too few to permit a meaningful investigation of predictors.

