# Supplementary material for: Reporting Bias in Drug Trials Submitted to the Food and Drug Administration: Review of Publication and Presentation
Source: PLoS Med. 2008 Nov 25;5(11):e217. doi: 10.1371/journal.pmed.0050217 (PMC2586350; doi:10.1371/journal.pmed.0050217)
Supplement: Table S1 — (78 KB DOC) [file pmed.0050217.st001.doc]

**Table S1: List of NDAs**

|  | Generic Name | Applicant | Approval Date | # NDA trials | # Published trials |
| --- | --- | --- | --- | --- | --- |
|  | Eplerenone | GD Searle | 09/27/02 | 13 | 8 |
|  | Ezetimibe | MSP Singapore | 10/25/02 | 12 | 12 |
|  | Eletriptan hydrobromide | Pfizer | 12/26/02 | 10 | **7 |
|  | Olmesartan medoxomil | Sankyo Pharma | 04/25/02 | 10 | 5 |
|  | Formoterol fumarate | Novartis | 02/16/01 | 9 | 9 |
|  | Galantamine hydrobromide | Janssen Research Foundation | 02/28/01 | 8 | 5 |
|  | Ertapenem sodium | Merck and Co, Inc | 11/21/01 | 7 | 7 |
|  | Aripiprazole | Otsuka America Pharm | 11/15/02 | 6 | **4 |
|  | Bimatoprost opthalmic solution | Allergan | 03/16/01 | 6 | 4 |
|  | Cefditoren pivoxil | TAP Holdings, Inc | 08/29/01 | 6 | 5 |
|  | Frovatriptan succinate | Vanguard | 11/08/01 | 6 | 5 |
|  | Atomoxetine HCl | Lilly | 11/26/02 | 6 | 6 |
|  | Almotriptan malate | Pharmacia & Upjohn | 05/07/01 | 6 | 5 |
|  | Ziprasidone | Pfizer Inc | 02/05/01 | 5 | 3 |
|  | Travoprost ophthalmic solution | Alcon Universal, Ltd | 03/16/01 | 5 | 4 |
|  | Fondaparinux sodium injection (Org31540/SR90107A) | Fonda BV | 12/07/01 | 5 | 5 |
|  | Pimecrolimus | Novartis Pharmaceuticals Corporation | 12/13/01 | 4 | 2 |
|  | Nitazoxanide | Romark Laboratories | 11/22/02 | 4 | 4 |
|  | Sodium oxybate | Orphan Medical Inc | 07/17/02 | 4 | 4 |
|  | Desloratadine | Schering | 12/21/01 | 4 | 2 |
|  | Nesiritide | Scios Inc | 08/10/01 | 4 | 4 |
|  | Dutasteride | GlaxoSmithKline | 11/20/01 | 3 | *0 |
|  | 7.5% Icodextrin Peritoneal Dialysis Solution | Baxter Healthcare Corporation | 12/20/02 | 3 | 3 |
|  | Treprostinil sodium | United Therapeutics | 05/21/02 | 2 | 2 |
|  | Bosentan | Actelion | 11/20/01 | 2 | 2 |
|  | Perflutren | Bristol Myers Squibb | 07/31/01 | 2 | 0 |
|  | Norelgestromin/ethinyl estradiol | R.W. Johnson Pharmaceutical Research Inst. | 11/20/01 | 2 | 1 |
|  | Drospirenone 3 mg and  ethinyl estradiol 0.030 mg | Berlex | 05/11/01 | 2 | 2 |
|  | Adefovir dipivoxil | Gilead Sciences | 09/20/02 | 2 | 2 |
|  | Fulvestrant | AstraZeneca | 04/25/02 | 2 | 2 |
|  | Tenofovir disoproxil fumarate (DF) | Gilead Sciences | 10/26/01 | 2 | 2 |
|  | Zoledronic acid | Novartis | 08/20/01 | 1 | 1 |
|  | Voriconazole | Pfizer Inc | 05/24/02 | 1 | 1 |
|  |  |  |  |  |  |
| EXCLUDED- Off market |  |  |  |  |  |
|  | Valdecoxib | GD Searle Corp | 11/16/01 |  |  |
|  | Tegaserod maleate | Novartis | 07/24/02 |  |  |

|  |  |  |  |  |  |
| --- | --- | --- | --- | --- | --- |
| EXCLUDED- No eligible trials |  |  |  |  |  |
|  | Caspofungin acetate for intravenous injection | Merck & Co, Incorporated | 01/26/01 |  |  |
|  | Oxaliplatin/5-FU/leucovorin | Sanofi-Synthelabo | 08/09/02 |  |  |
|  | Etonogestrel; ethinyl estraiol vaginal ring | Organon | 10/03/01 |  |  |
|  | Imatinib mesylate | Novartis | 05/10/01 |  |  |
|  | Dimyristoylphosphatidylcholine; perflexane | Alliance Pharm | 05/31/02 |  |  |
|  | Nitisinone | Swedish Orphan | 01/18/02 |  |  |

* The trial data were reported individually for each trial to the FDA, but were pooled together for publication. There was no publication of data from an individual trial.

** 1 in press
